# Supplementary material for: Alterations of the Gut Microbiome in Chronic Hepatitis B Virus Infection Associated with Alanine Aminotransferase Level
Source: J Clin Med. 2019 Feb 2;8(2):173. doi: 10.3390/jcm8020173 (PMC6407135; doi:10.3390/jcm8020173)
Supplement: Supplementary file 1 [file jcm-08-00173-s001.pdf]

Table S1. Pairwise comparison of alpha diversity by Kruskal-Wallis test.

	Group 1	Group 2	H	*p*-Value	*q*-Value
Faith PD	Control	Normal ALT	2.000	0.157	0.399
		High ALT	1.237	0.265	0.399
	Normal ALT	High ALT	0.006	0.939	0.939
Evenness	Control	Normal ALT	1.392	0.238	0.238
		High ALT	2.052	0.152	0.228
	Normal ALT	High ALT	4.380	0.036	0.109
Shannon	Control	Normal ALT	1.341	0.247	0.337
		High ALT	0.922	0.337	0.337
	Normal ALT	High ALT	2.932	0.087	0.261
Observed OTUs	Control	Normal ALT	1.693	0.193	0.565
		High ALT	0.782	0.377	0.565
	Normal ALT	High ALT	0.093	0.761	0.761

Table S2. Pairwise comparison of beta diversity by PERMANOVA test.

	Group 1	Group 2	Pseudo-F	*p*-Value	*q*-Value
Unweighted UniFrac	Control	Normal ALT	0.888	0.531	0.651
		High ALT	0.917	0.526	0.651
	Normal ALT	High ALT	0.815	0.651	0.651
Weighted UniFrac	Control	Normal ALT	0.676	0.596	0.596
		High ALT	2.815	0.041	0.062
	Normal ALT	High ALT	3.648	0.013	0.039

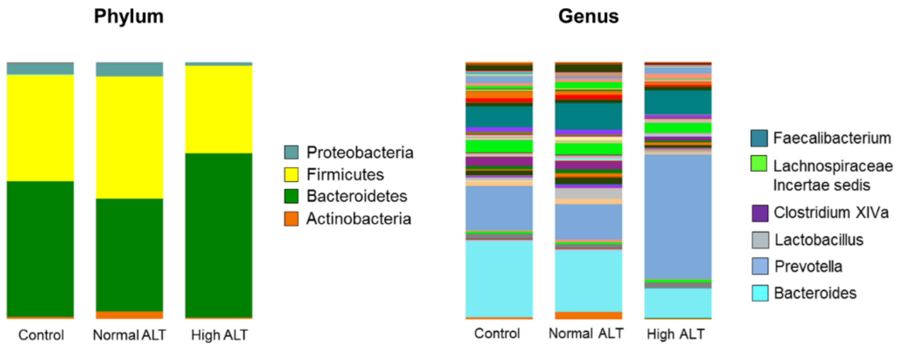

Figure S1. Bar chart of the proportional abundance at phylum and genus levels in three categories.
